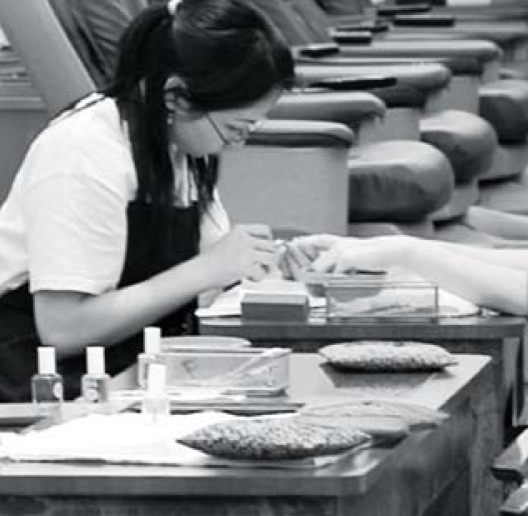# The Beat

**Published:** 2008-07

**Authors:** Erin E. Dooley

## U.S. Pesticide Survey Shelved

The USDA announced in May 2008 that it was suspending publication of the annual *Agricultural Chemical Usage* reports until at least 2010 because of budget constraints. Published since 1990, the reports have tracked the amount of pesticides applied to crops nationwide, providing information to stakeholders such as pesticide manufacturers and consumer groups as well as the U.S. EPA, which uses the reports when evaluating pesticides for regulation. According to the USDA, information in the reports can still be obtained from private sources, but in an open letter to the department secretary, farm and environmental groups wrote that purchasing such data is prohibitively expensive—as much as $500,000—whereas the high-quality data gathered by the government was free to everyone.

## Invasion of the Biocrops?

To address energy shortages and the impact of climate change, the world’s biofuel industries are searching for fast-growing high-yield crops to turn into plant-based fuels. In a May 2008 report titled *Biofuel Crops and the Use of Non-Native Species: Mitigating the Risks of Invasion*, the Global Invasive Species Programme (GISP) warns that importing such crops for use as feedstocks runs the risk of releasing invasive species, adding to a global biodiversity threat that currently costs an estimated $1.4 trillion annually to fight. In the report GISP sets forth recommendations on risk assessment and management, cost–benefit analysis, and certification and accreditation processes to avoid negative impacts from potentially invasive biocrop species. The report also provides a list of biofuel species classified according to their potential risk.

**Figure:**
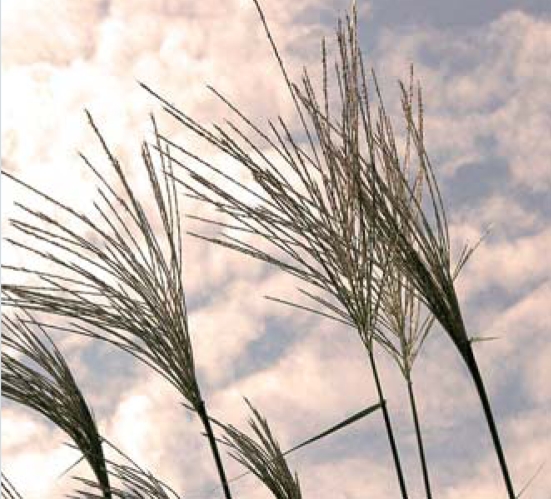
Miscanthus x giganteus

## Persistent Pesky Dust Mites

According to a 54-study review published in issue 2 (2008) of the *Cochrane Database of Systematic Reviews*, despite all we do to rid our homes of dust mites, these pesky arthropods continue to thrive in carpet, bedding, and other fabric-covered items and produce allergens that can cause bronchial distress and other allergy symptoms in sensitive individuals. Such individuals are often advised to purchase special bedding and cleaning devices and to follow exhaustive home maintenance regimens to control dust mites. Yet the review authors conclude that such steps do little to reduce exposures—probably because the levels of allergens in most homes are high enough that even a 90% reduction leaves enough allergen to trigger symptoms.

## Stone Zone Expansion

The U.S. Southeast is sometimes referred to as the Stone Belt because of a higher incidence of kidney stones, possibly due to dehydration in the warmer climate as well as diets rich in salt, meat, tea, and other foods linked to the condition. Research published in the April 2008 issue of the *Journal of Urology* warns that higher temperatures caused by climate change could expand the Stone Belt, putting an additional 10% of the U.S. population in high-risk “stone zones” by 2050. This expansion could translate to an increase of 1 to 2 million lifetime cases of kidney stone disease, with treatment costs climbing as high as $1 billion annually—a 10–20% increase over current estimates.

**Figure:**
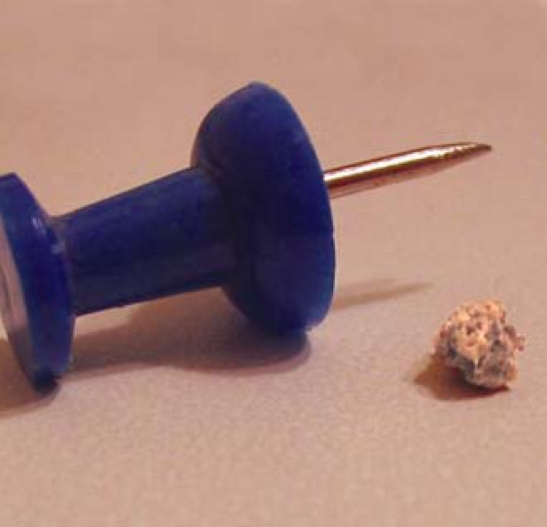
Kidney stones are concretions of minerals from urine

## Quick Hit to the Vascular System

Although governments around the world are are banning smoking in public areas in response to warnings of scientists about the harmful health effects of secondhand smoke (SHS), many people are still exposed daily to SHS, even if only for brief periods. A new study published in the 6 May 2008 issue of the *Journal of the American College of Cardiology* suggests that exposure to SHS in real-world settings for as little as 30 minutes can adversely affect the body’s vascular system, even in young and otherwise healthy lifelong non-smokers. The harm was twofold: SHS exposure not only injured blood vessels but also impaired the ability of endothelial progenitor cells to repair the damaged vessels. The study further showed that the harmful response to SHS exposure persisted for at least 24 hours, much longer than previously believed.

## CA Nail Salon Workers Surveyed

Most of the 35,000 nail salons in California employ Asian immigrants who are routinely exposed to potentially harmful compounds in nail care products—some of which are unregulated—including carcinogens and endocrine disruptors. In a study published online 14 May 2008 ahead of print in the *Journal of Community Health*, investigators collected preliminary descriptive data from Vietnamese nail salon workers in Alameda County to help inform future targeted health interventions and reduce occupational exposure. A majority of the workers expressed concern about chemical exposures in the workplace, and many reported acute symptoms such as skin and eye irritation, breathing difficulties, and headaches associated with solvent exposure. The authors note that although nail care products are thought to contain low levels of harmful compounds, their prevalence in salons means exposure is likely continuous, and the chronic effects are unknown. The U.S. EPA and partner agencies are reaching out to salon workers, many of whom do not speak English. The 2007 report *Protecting the Health of Nail Salon Workers*, which offers guidance on minimizing exposures, is available in English, Vietnamese, and Korean.

**Figure:**